# Combination Therapy with Human Chorionic Villi MSCs and Secretory Factors Enhances Cutaneous Wound Healing in a Rat Model

**DOI:** 10.3390/ijms26146888

**Published:** 2025-07-17

**Authors:** Qingwen Deng, Jiawei Huang, Lai Ling Tsang, Jinghui Guo, Chi Chiu Wang, Xiaohu Zhang, Xiaohua Jiang

**Affiliations:** 1 School of Biomedical Sciences, Faculty of Medicine, The Chinese University of Hong Kong, Hong Kong SAR, China; 1155210684@link.cuhk.edu.hk (Q.D.); 1155191743@link.cuhk.edu.hk (J.H.); angeltsang@cuhk.edu.hk (L.L.T.); 2 School of Medicine, The Chinese University of Hong Kong, Shenzhen 518172, China; guojinghui@cuhk.edu.cn; 3 Department of Obstetrics and Gynaecology, Faculty of Medicine, The Chinese University of Hong Kong, Hong Kong SAR, China; ccwang@cuhk.edu.hk; 4 Reproduction and Development, Li Ka Shing Institute of Health Sciences, The Chinese University of Hong Kong, Hong Kong SAR, China; 5 Sichuan University-The Chinese University of Hong Kong Joint Laboratory for Reproductive Medicine, West China Second University Hospital, Sichuan University, Chengdu 610041, China; zhangxiaohu@scu.edu.cn; 6 Shenzhen Research Institute, The Chinese University of Hong Kong, Shenzhen 518000, China; 7 CUHK-GIBH CAS Joint Research Laboratory on Stem Cell and Regenerative Medicine, The Chinese University of Hong Kong, Hong Kong SAR, China

**Keywords:** wound healing, chorionic villus, MSCs, secretome, combination therapy

## Abstract

Cutaneous wound healing is a complex process involving multiple cellular and molecular events, and current treatments often face limitations in efficacy and safety. Stem-cell therapy, particularly using mesenchymal stem cells (MSCs), has emerged as a promising approach to enhance wound repair through both direct cell replacement and paracrine signaling. This study investigates the therapeutic potential of human chorionic villus mesenchymal stem cells (hCV-MSCs) and their secretory factors in enhancing cutaneous wound healing. Utilizing a rat model, we combined the local administration of hCV-MSC-laden PEGDA/SA/Col-I hydrogel with the systemic delivery of their secretome, aiming to leverage the complementary mechanisms of cellular and cell-free therapies. Our findings demonstrate that hCV-MSCs delivered via PEGDA/SA/Col-I hydrogel significantly accelerated wound closure compared to controls, with near-complete closure observed by day 20. Histological analysis revealed enhanced keratinocyte maturation (increased KRT10/KRT14 ratio) and a higher density of CD31^+^ blood vessels, indicating improved re-epithelialization and angiogenesis. A mass spectrometry analysis of the hCV-MSC secretome identified 849 proteins, with enrichment in pathways related to ECM organization, cell adhesion, and immune regulation. Key proteins such as ANXA1, SERPINE1, and WNT5A were implicated in wound-healing processes. Combination therapy with systemic secretome administration further accelerated wound closure and enhanced collagen deposition, keratinocyte maturation, and vascularization compared to hCV-MSCs alone. Our results highlight the promising application of hCV-MSCs and their secretome in cutaneous wound healing, paving the way for innovative therapeutic strategies that integrate both local and systemic regenerative approaches.

## 1. Introduction

Cutaneous wound healing is a complex and dynamic process involving a series of highly coordinated cellular and molecular events aiming at restoring the structural integrity and functional capacity of damaged skin tissue [1]. This intricate process encompasses four overlapping and interdependent phases: hemostasis, inflammation, proliferation, and remodeling. Initially, during the hemostasis phase, vasoconstriction and platelet aggregation rapidly occur, forming a fibrin clot that provides protection and serves as a scaffold facilitating subsequent cellular infiltration. This is rapidly followed by the inflammatory phase, characterized by the infiltration and activation of immune cells, including neutrophils and macrophages, which eliminate pathogens, clear cellular debris, and secrete cytokines critical for initiating tissue repair. During the proliferative phase, keratinocytes, fibroblasts, and endothelial cells proliferate and migrate into the wound bed, resulting in re-epithelialization, granulation tissue formation, and angiogenesis [2,3]. Lastly, the remodeling phase involves the gradual maturation, realignment, and reorganization of collagen fibers within the extracellular matrix (ECM), leading to wound contraction and restoration of skin tissue strength and functionality [4]. Each stage of cutaneous wound healing is tightly regulated by numerous signaling molecules, including growth factors, cytokines, chemokines, and matrix metalloproteinases [4,5]. Despite advances in our understanding of cutaneous wound healing, current therapeutic strategies—such as surgical approaches, negative-pressure wound therapy, topical antimicrobial and antiseptic treatments, dressings, and growth factor-based therapies—often encounter limitations including donor-site morbidity, infection risks, high costs, and variable efficacy, particularly in chronic or extensive wounds [6,7]. Therefore, there remains an urgent clinical need for novel therapeutic modalities that can enhance cutaneous wound repair and improve overall functional and cosmetic outcomes.

Over the past few decades, stem-cell therapy has emerged as a promising approach to address the complex pathological mechanisms underlying impaired cutaneous wound healing. Among various stem-cell types, mesenchymal stem cells (MSCs) have attracted significant attention due to their advantageous biological properties, including ease of isolation, minimal ethical concerns, immunomodulatory capabilities, and low immunogenicity. Numerous preclinical studies have demonstrated that exogenously administered MSCs can effectively mitigate local and systemic inflammatory responses, activate anti-apoptotic signaling pathways, promote tissue regeneration, and establish a supportive microenvironment conducive to endogenous repair [8,9,10]. Although the precise cellular and molecular mechanisms underlying MSC-mediated therapeutic effects remain incompletely understood, accumulating evidence strongly suggests that their beneficial effects are primarily attributed to paracrine signaling [11,12]. MSCs possess a remarkable capacity to secrete a diverse array of bioactive molecules—including growth factors, cytokines, chemokines, and ECM proteins—which collectively orchestrate critical events within the wound-healing cascade [13,14,15]. Recent advances have further emphasized the therapeutic potential of the MSC-derived secretome, defined as the complete repertoire of bioactive molecules secreted by MSCs [16]. The MSC secretome has shown significant promise in accelerating tissue regeneration, modulating inflammatory responses, enhancing angiogenesis, and minimizing scar formation [17,18,19]. As a cell-free therapeutic strategy, secretome-based therapy offers distinct advantages by circumventing potential limitations associated with direct cell transplantation, such as immunological reactions, tumorigenicity risks, and logistical challenges, thereby providing a safer, more efficient, and clinically applicable alternative for enhancing cutaneous wound healing and skin regeneration [17,20,21].

Despite the promising therapeutic potential of MSCs, their clinical translation has been hindered by several challenges, including limited proliferative capacity and cellular senescence following prolonged in vitro culture [22,23]. Additionally, MSCs derived from adult tissues at higher passages are more likely to induce immune responses upon transplantation, potentially affecting clinical efficacy [24]. To overcome these limitations, MSCs derived from perinatal tissues have emerged as attractive alternatives, offering advantages of abundant availability, ease of collection, strong proliferative potential, favorable immunomodulatory properties, and minimal ethical concerns [25,26]. Among these perinatal sources, human chorionic villi-derived MSCs (hCV-MSCs) have demonstrated particular promise due to their exceptional proliferative ability, robust immunomodulatory effects, and enhanced differentiation capacity, compared to other placental MSC populations [27,28,29,30,31]. Our previous studies demonstrated that MSCs derived from perinatal tissues significantly accelerated cutaneous wound healing in diabetic db/db mouse models [32]. Building upon this foundation, the current study aims to investigate the therapeutic potential of hCV-MSCs and their secretory factors in promoting cutaneous wound healing using a rat model. Specifically, we hypothesize that the local transplantation of hCV-MSCs, combined with the systemic administration of their secretome, may synergistically enhance the regenerative process, resulting in improved wound closure, enhanced re-epithelialization, and increased angiogenesis.

## 2. Results

### 2.1. hCVMSCs Enhance Cutaneous Wound Healing in a Rat Model

Three independent hCVMSC lines were successfully established as described in the Materials and Methods (Figure S1A). MTT assays performed at passage 3 confirmed high proliferative capacity (Figure S1B). Flow cytometry analysis revealed a strong expression of MSC markers (CD90, CD105, CD73, CD44) and an absence of hematopoietic markers (CD34, CD11b, CD19, CD45, and HLA-DR), consistent with the MSC phenotype (Figure S2A). Trilineage differentiation potential was confirmed by Alizarin Red, Oil Red O, and Alcian Blue staining, indicating osteogenic, adipogenic, and chondrogenic differentiation, respectively (Figure S2B).

To assess the therapeutic potential of hCVMSCs in cutaneous wound healing, full-thickness dorsal wounds (10 mm in diameter) were created in SD rats. Compared to mice, rats exhibit a slower wound contraction rate and a healing process that more closely resembles human cutaneous wound healing, making them a more suitable model for evaluating therapeutic interventions. Building on our previous study, which demonstrated the biocompatibility and supportive properties of PEGDA/SA/Col-I hydrogel in promoting diabetic wound healing [32], hCVMSCs were encapsulated within this matrix for in vivo delivery.

In vitro viability assays confirmed high cell survival within the hydrogel, along with sustained proliferation over seven days (Figure S3A,B), further underscoring the hydrogel’s excellent biocompatibility. Furthermore, the secretory function of hCVMSCs was validated, as over 200 µg of proteins were secreted by the encapsulated cells, demonstrating their robust secretory capability (Figure S3C).

**Figure 1:**
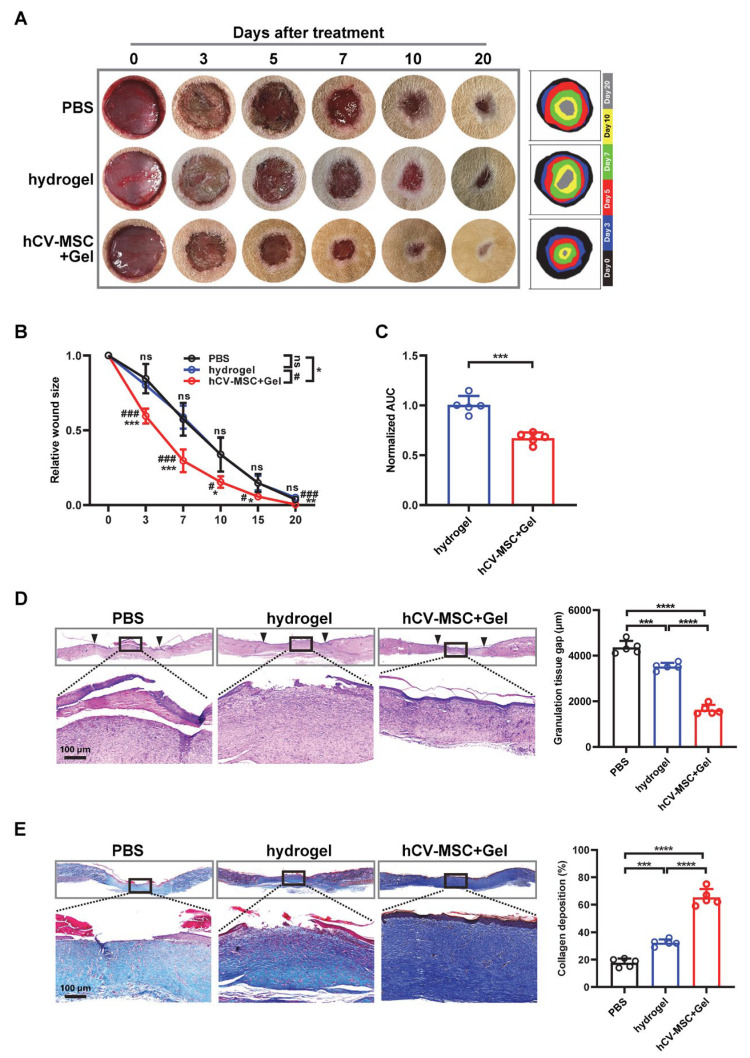
hCV-MSCs enhance cutaneous wound healing in rats. (**A**) Representative photographs of full-thickness excision wounds at 0, 3, 5, 7, 10, and 20 days post-wounding. Dynamic traces of wound sites are shown on the right. (**B**) Relative wound sizes are expressed as percentages of initial wound area, with n = 5 for all groups. Data are presented as mean ± SD. * *p* < 0.05, ** *p* < 0.01 and *** *p* < 0.001 indicate significance compared to the PBS group; # *p* < 0.05 and ### *p* < 0.001 denote comparisons between hCV-MSCs and hydrogel-only groups; ns represents no significance. Statistical significance was determined using Tukey’s post hoc test following one-way ANOVA (*p* < 0.05). (**C**) Mean area under the curve (AUC) for individual wounds is shown, n = 5 for all groups. AUC values were normalized to the mean AUC of vehicle controls. Data are presented as mean ± SD. *** *p* < 0.001 by Student’s *t*-test. (**D**) Hematoxylin and eosin (H&E) staining of wounds on day 20 for each group. The quantification of granulation tissue gaps on day 20 is shown on the right; n = 5 for all groups. Data are presented as mean ± SD. *** *p* < 0.001 and **** *p* < 0.0001 by Tukey’s post hoc test when significant by one-way ANOVA (*p* < 0.05). (**E**) Masson’s trichrome staining (MTS) of wounds on day 20. The quantification of collagen deposition on day 20; n = 5 for all groups. Data are presented as mean ± SD. *** *p* < 0.01 and **** *p* < 0.001 by Tukey’s post hoc test when significant by one-way ANOVA (*p* < 0.05).

Subsequently, wounds were treated with PBS (vehicle control), hydrogel alone, or hCVMSC-laden hydrogel immediately post-injury. Macroscopic evaluation revealed accelerated wound closure in the hCVMSC-treated group compared to vehicle controls (Figure 1A). A quantitative analysis of the wound area over time showed significant differences beginning on day 3, with near-complete closure observed by day 20 in the hCVMSC group (Figure 1B). By comparison, residual wound areas in the PBS and hydrogel groups were 3.7 ± 1.9% and 5.0 ± 1.4%, respectively. To further evaluate the therapeutic efficacy, the area under the curve (AUC) for wound area over time was calculated and normalized to vehicle control. The hCVMSC group exhibited a significantly lower AUC compared to the hydrogel groups (0.671 ± 0.056 vs. 1.004 ± 0.091), indicating enhanced wound resolution (Figure 1C). In line with the observed rates of wound closure, histological analysis on day 20 revealed a reduced granulation tissue gap in the hCVMSC-treated group compared to both control groups (Figure 1D). Quantitative analysis confirmed significant differences between all groups. Masson’s trichrome staining demonstrated increased collagen deposition in the hydrogel and hCVMSC groups, with the most pronounced effect observed in the hCVMSC group (Figure 1E). Collectively, these results demonstrate that hCVMSCs, delivered via a PEGDA/SA/Col-I hydrogel, significantly enhance wound healing by accelerating closure, reducing granulation tissue gap, and promoting collagen deposition.

### 2.2. hCVMSCs Promote Re-Epithelization and Angiogenesis While Ameliorating Inflammation

**Figure 2:**
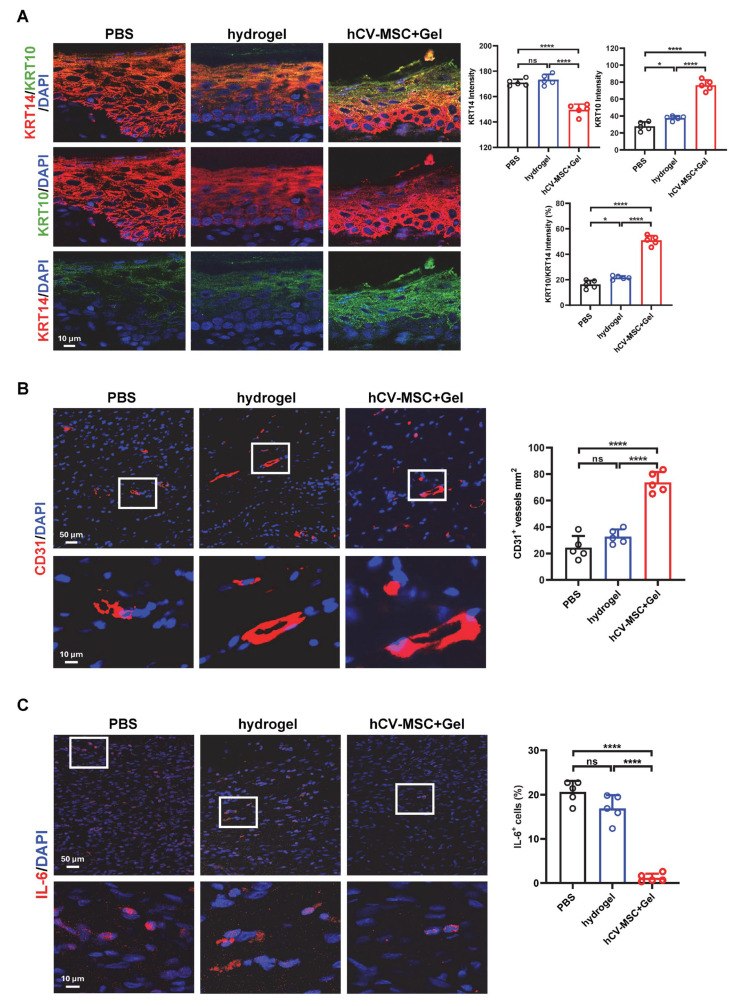
hCV-MSCs promote re-epithelization and angiogenesis. (**A**) Representative IF images of KRT10 and KRT14 staining in wounds on day 20 for each experimental group. The quantification of the average intensity percentages of KRT10^+^, KRT14^+^, and the KRT10^+^/KRT14^+^ ratio is shown on the right. (**B**) Representative IF images of CD31 staining in wounds on day 20 for each experimental group. White boxes outline the enlarged areas. The quantification of CD31^+^ cells per unit area is presented on the right. (**C**) Representative IF images of IL-6 staining in wounds on day 20 for each experimental group. White boxes outline the enlarged areas. The quantification of IL-6^+^ cell percentage is shown on the right. All data are presented as mean ± SD, with n = 5 for all groups. * *p* < 0.05 and **** *p* < 0.0001, and ns represents no significance, as determined by Tukey’s post hoc test following one-way ANOVA (*p* < 0.05).

Re-epithelialization is a critical step in wound healing, characterized by the migration, proliferation, and differentiation of epidermal keratinocytes to restore the skin barrier [33]. To assess keratinocyte behavior during wound healing, we evaluated the expression of Keratin 14 (KRT14) and Keratin 10 (KRT10), markers of immature and mature keratinocytes, respectively. IF analysis revealed significantly higher levels of KRT10 expression in the hCVMSC-treated group compared to the hydrogel control, indicating enhanced keratinocyte differentiation. Furthermore, the ratio of KRT10 to KRT14 intensity—a marker of keratinocyte maturation—was significantly increased in the hCVMSC-treated group, underscoring the role of hCVMSCs in accelerating epidermal maturation and re-epithelialization (Figure 2A). Angiogenesis is another essential component of wound healing, providing oxygen and nutrients necessary for tissue repair. IF staining for CD31, a marker of endothelial cells, demonstrated a higher density of CD31^+^ blood vessels in the hCVMSC-treated group compared to the hydrogel control. These findings confirm that hCVMSCs significantly promote neovascularization within the wound bed, contributing to enhanced tissue regeneration (Figure 2B). In addition to promoting re-epithelialization and angiogenesis, hCVMSCs demonstrated an anti-inflammatory effect during the later stages of wound healing. On day 20, wounds treated with hCVMSCs exhibited markedly reduced expression of interleukin-6 (IL-6), a key pro-inflammatory cytokine, compared to the hydrogel control. This suppression of IL-6 suggests that hCVMSCs help resolve inflammation, facilitating the transition to the remodeling phase of wound healing. Collectively, these results demonstrate that hCVMSCs enhance cutaneous wound healing by promoting keratinocyte maturation and re-epithelialization, stimulating angiogenesis, and ameliorating inflammation during the resolution phase.

### 2.3. Analysis of Secretome Profiles of hCVMSCs Reveals Biological Pathways Associated with Wound Healing and Inflammatory Response

**Figure 3:**
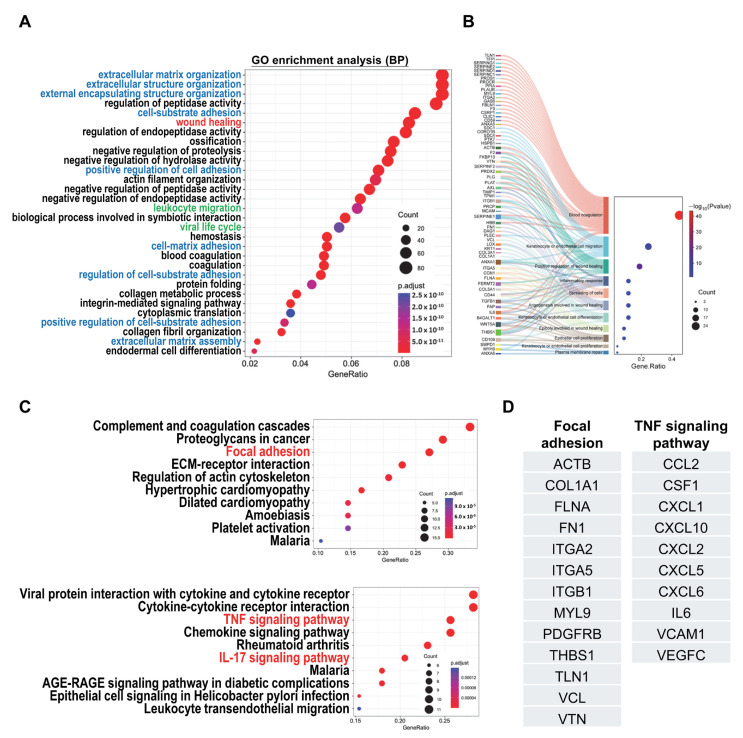
An analysis of the secretome profiles of hCVMSCs. (**A**) GO enrichment analysis of the 849 identified proteins. GOs related to ECM and cell–substrate adhesion are highlighted in blue, GOs related to wound healing are highlighted in red, and GOs related to immune regulation are highlighted in green. (**B**) A Sankey dot plot illustrating biological processes associated with the 69 proteins linked to wound healing. (**C**) KEGG pathway analysis of the 69 proteins involved in wound healing and 52 proteins related to immune regulation. (**D**) A list of proteins identified within the focal adhesion and TNF-α pathways.

The therapeutic efficacy of MSCs is primarily attributed to their paracrine effects. Our previous study demonstrated that the secretome derived from hCVMSCs promotes keratinocyte and endothelial cell proliferation and migration [32]. To further understand the regulatory effects of hCVMSC-derived secretory factors, we performed mass spectrometry to analyze the protein composition of the secretome. A total of 849 proteins were identified, and gene ontology (GO) analysis revealed enrichment in processes such as extracellular matrix (ECM) organization, cell–matrix adhesion, wound healing, and immune regulation (Figure 3A). The substantial array of secretory proteins involved in extracellular matrix (ECM) organization and cell–substrate adhesion highlights the identity of hCV-MSCs, characterized by a robust ECM composition that supports essential processes such as cell adhesion, migration, and intercellular communication. These mechanisms are particularly vital during placental development. To explore the molecular mechanisms underlying the therapeutic effects of hCVMSCs, we identified 69 proteins specifically associated with wound healing. These proteins were involved in processes such as blood coagulation, keratinocyte/endothelial cell migration, inflammatory response, angiogenesis, cell spreading, and cell differentiation/proliferation (Figure 3B). Notably, key proteins, including ANXA1 (Annexin A1), SERPINE1 (PAI-1), TGFB1 (TGF-β1), IL-6 (interleukin-6), WNT5A, and THBS1 (Thrombospondin-1), were implicated in multiple roles during wound healing. Further KEGG pathway analysis on wound healing and leukocyte migration highlighted the involvement of the focal adhesion pathway and the TNF-α signaling pathway (Figure 3C). Noteworthy proteins in the focal adhesion pathway included COL1A1 (collagen I A1), fibronectin (FN1), integrins, TLN1 (Talin-1), VCN (vinculin), and THBS1. Additionally, various chemokines and cytokines—such as CCL2 (C-C motif chemokine ligand 2), CXCL1 (C-X-C motif chemokine ligand 1), CXCL10 (C-X-C motif chemokine ligand 10), IL-6 (C-X-C motif chemokine ligand 10), and VCAM1 (vascular cell adhesion molecule 1)—were identified as key mediators of the TNF-α pathway (Figure 3D).

### 2.4. Therapeutic Effects of hCVMSC-Laden Hydrogel and Secretome Administration on Wound Healing

**Figure 4:**
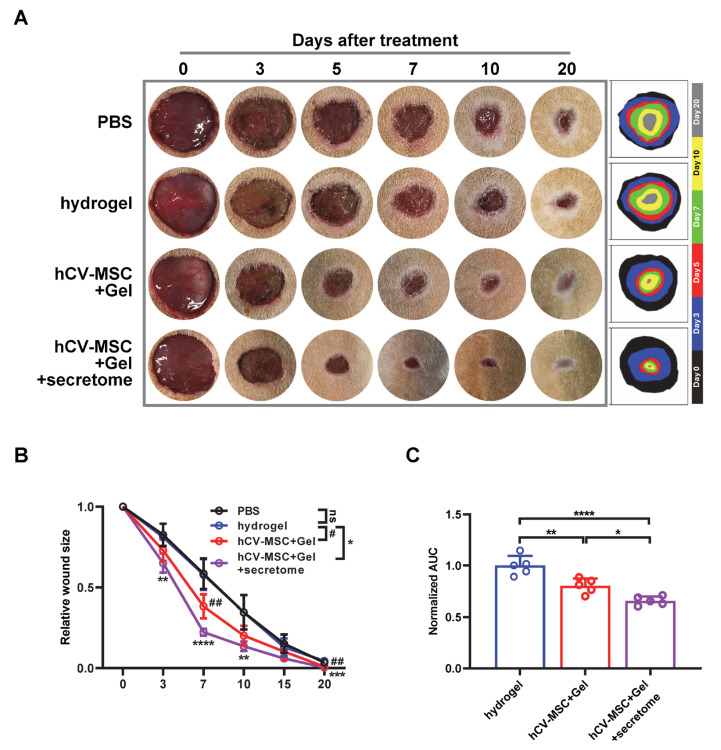
Combination therapy with secretome enhances wound healing. (**A**) Representative photographs of full-thickness excision wounds at 0, 3, 5, 7, 10 and 20 days after wounding. Dynamic traces of wound sites are shown on the right. (**B**) The relative wound size is shown for each group, with n = 5 for all groups. Wound areas are normalized to the original wound size and expressed as the percentage of wound closure versus initial wound size. All data are presented as mean ± SD. *, **, ***, and **** represent *p* < 0.05, 0.01, 0.001, and 0.0001, respectively, for comparisons between hCV-MSC + Gel + secretome group and hydrogel group. The symbols # and ## represent *p* < 0.05 and 0.01, for comparisons between the hCV-MSC + Gel group and hydrogel group and ns represents no significance for comparison between PBS and hydrogel group. Statistical significance is determined using Tukey’s post hoc test following a one-way ANOVA (*p* < 0.05). (**C**) Mean area-under-curve (AUC) of individual wounds of each group, n = 5 for all groups. The AUC of individual wounds in treatment groups was normalized against the mean AUC of vehicle controls within the same experimental runs. All data are presented as mean ± SD. *, **, and **** represent *p* < 0.05, 0.01, and 0.0001, respectively, by Tukey’s post hoc test when statistical significance by one-way ANOVA (*p* < 0.05) is obtained.

**Figure 5:**
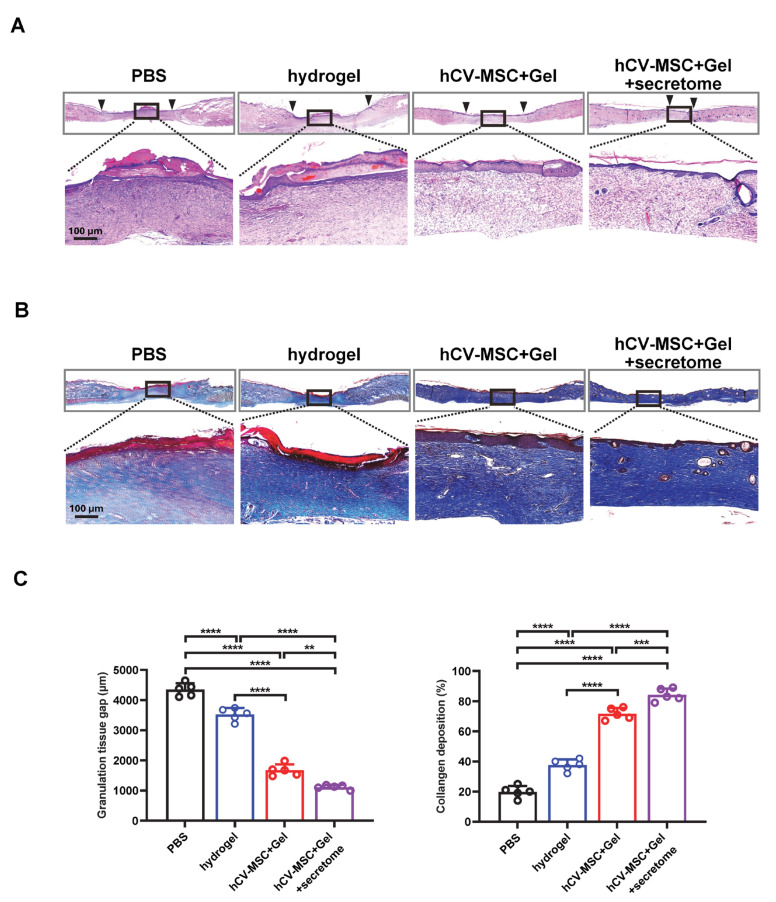
Combination therapy with secretome reduces granulation tissue gap and increases collagen deposition. (**A**) H&E staining of wounds on day 20 for each group. (**B**) Masson’s trichrome staining (MTS) of wounds on day 20 μm. (**C**) The quantification of (**A**,**B**), n = 5 for all groups. Data are presented as mean ± SD. ** *p* < 0.01, *** *p* < 0.001 and **** *p* < 0.0001 by Tukey’s post hoc test when significant by one-way ANOVA (*p* < 0.05).

**Figure 6:**
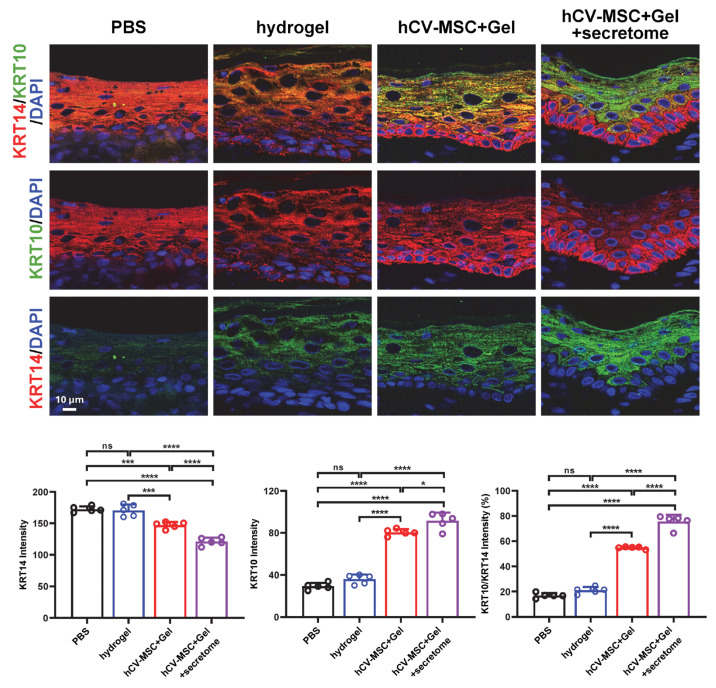
Combination therapy with secretome promotes keratinocyte maturation. Representative IF images of KRT10 and KRT14 staining in wounds on day 20 for each experimental group. The quantification of the average intensity percentage of KRT10, KRT 14, and KRT10/KRT14 is shown below. n = 5 for all groups. Data are presented as mean ± SD. * *p* < 0.05, *** *p* < 0.001 and **** *p* < 0.0001 and ns represents no significance by Tukey’s post hoc test when significant by one-way ANOVA (*p* < 0.05).

**Figure 7:**
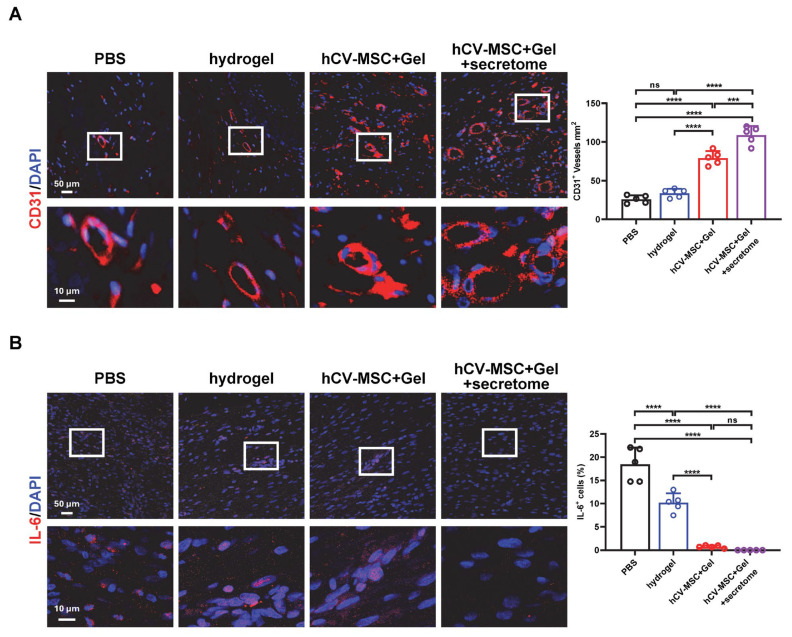
Combination therapy with secretome promotes angiogenesis. (**A**) Representative IF images of CD31 staining in wounds on day 20 for each experimental group. White boxes outline the enlarged areas. The quantification of CD31^+^ cells per unit area is shown on the right. All data are presented as mean ± SD, n = 5 for all groups. *** and **** represent *p* < 0.001 and 0.0001, respectively, ns represents no significance by Tukey’s post hoc test when statistical significance by One-way ANOVA (*p* < 0.05) is obtained. (**B**) Representative IF images of IL-6 staining in wounds on day 20 for each experimental group. White boxes outline the enlarged areas. Quantification of IL-6^+^ cell percentage is shown on the right. All data are presented as mean ± SD, with n = 5 for all groups. **** *p* < 0.0001, and ns represents no significance by Tukey’s post hoc test following one-way ANOVA (*p* < 0.05).

Given the significant wound-healing and immune regulatory properties of the hCVMSC secretome, we evaluated the therapeutic effects of hCVMSC-laden PEGDA/SA/Co-I hydrogel, combined with systemic secretome administration, to enhance therapeutic efficacy. Compared to the vehicle and hydrogel-only groups, wounds treated with hCVMSCs exhibited a consistent, accelerated reduction in wound size (Figure 4A). Notably, combination therapy with systemic secretome administration resulted in a more significant acceleration of healing (Figure 4A,B). The combination therapy demonstrated a significantly smaller AUC compared to hCVMSCs alone (Figure 4C). Additionally, wounds treated with combination therapy exhibited decreased granulation tissue gaps and a higher intensity of collagen fibers in the dermal connective tissue compared to those treated with hCVMSCs only, indicating enhanced re-epithelialization and improved healing (Figure 5A–C). Consistently, combination therapy also resulted in a significant increase in KRT10 intensity and the KRT10–KRT14 ratio, indicative of improved epidermal differentiation (Figure 6). Enhanced vascularization was more evident in the combination therapy group, as demonstrated by a higher density of CD31^+^ vessels compared to the hCVMSC-only group (Figure 7A). Finally, while hCVMSCs alone significantly suppressed IL-6 expression in the healed wounds, no further reduction was observed with systemic secretome administration (Figure 7B). Collectively, these findings highlight the synergistic effects of secretome administration with hCVMSC-laden hydrogel, promoting enhanced wound closure, re-epithelialization, collagen deposition, and angiogenesis.

## 3. Discussion

The human placenta, regarded as medical waste after delivery, has emerged as a valuable source of MSCs for regenerative medicine. Human placenta-derived MSCs (hPD-MSCs) have garnered significant attention due to their unique biological properties and ethical advantages [29]. Notably, hPD-MSCs can be categorized based on their origin within the placenta, revealing distinct characteristics observed between fetal and maternal sources [34,35]. For instance, fetal-derived hPD-MSCs, such as those from the chorionic villi, often exhibit superior proliferation and differentiation potential compared to their maternal counterparts [35]. Specifically, hCV-MSCs isolated from the innermost layer of the placenta play a crucial role in maternal–fetal nutrient and gas exchange [36]. They exhibit high proliferative capacity and robust multilineage differentiation potential, making them particularly suitable for regenerative applications. Additionally, hCV-MSCs possess strong immunomodulatory properties, essential for mitigating immune responses in therapeutic settings [29].

While direct evidence on the use of hCV-MSCs for skin wound healing is limited, our current study demonstrates that hCV-MSCs significantly enhance cutaneous wound healing in a rat model. Our findings indicate increased keratinocyte maturation, evidenced by a higher KRT10/KRT14 ratio, and enhanced angiogenesis, as shown by increased CD31+ microvessel density. Moreover, hCV-MSCs reduced pro-inflammatory IL-6 levels, suggesting their capacity to favorably modulate the inflammatory response. This work, consistent with our previous findings on diabetic wound healing [32], contributes to the growing body of evidence supporting the therapeutic potential of hCV-MSCs in injury repair and highlights their unique advantages in promoting cutaneous wound healing.

Our secretome profile of hCVMSCs has yielded valuable insights into the regulatory mechanisms associated with wound healing. An analysis of GO related to wound healing identified key proteins, such as ITGB1, SERPINE1, ANXA1, and WNT5A, which play critical roles in various cellular processes essential for effective wound healing. These processes include blood coagulation, the migration of keratinocytes and endothelial cells, inflammatory responses, angiogenesis, and cell proliferation and differentiation (Figure 3B). For instance, ITGB1 is a pivotal in wound healing, influencing cell adhesion, migration, signaling, angiogenesis, inflammation, and ECM remodeling. Its proper function is essential for efficient tissue regeneration and repair following injury [37,38]. Serpin E1, also known as plasminogen activator inhibitor-1 (PAI-1), is a multifunctional protein that significantly contributes to wound healing by regulating fibrinolysis and promoting cell migration and proliferation. Additionally, it modulates inflammation, supports angiogenesis, and facilitates collagen synthesis [39,40,41]. The balanced expression of PAI-1 is essential for effective tissue repair and regeneration, particularly through its role in activating host-tissue matrix metalloproteinases (MMPs), which are critical for restructuring the ECM architecture. This dynamic remodeling is vital for the mobilization of regenerating cells to the wound site, thereby creating an environment conducive to cell migration and tissue repair. Annexin A1 plays a multifaceted role in wound healing by regulating inflammation, facilitating cell migration and proliferation, modulating apoptosis, restoring epithelial barriers, and promoting angiogenesis. Its actions ensure a coordinated and efficient healing process, making it a crucial factor in tissue repair and regeneration [42,43]. WNT5A is another essential protein that significantly contributes to wound healing by promoting cell migration and proliferation, regulating angiogenesis, modulating inflammation, and remodeling the ECM [42,43]. It activates non-canonical Wnt signaling, which has been shown to play a critical role in corneal epithelial wound healing [44]. Furthermore, WNT5A has been implicated in regulating epithelial differentiation during cutaneous wound healing, further underscoring its multifaceted role in tissue repair [42]. Although our proteomic analysis identified WNT5A and other key proteins enriched in the hCVMSC secretome and associated with wound-healing pathways, their individual contributions were not validated. Such targeted analyses are essential to confirm their role in regeneration and warrant further investigation.

Focal adhesions are crucial for cell attachment and migration, mediating ECM interactions and associated signaling pathways [45,46]. Our analysis identified several key ECM proteins within the hCV-MSC secretome, including fibronectin (FN1), collagen I A1 (COL1A1), vitronectin (VTN), and vinculin (VCL). These proteins are essential for the formation and functionality of focal adhesions, which are crucial for effective cell migration and tissue repair [1,47]. In addition to ECM components, our findings revealed a group of cytokines involved in the TNF-α and IL-17 signaling pathways, which are vital for wound repair through the recruitment and modulation of immune cells, including neutrophils, macrophages, and T cells. Notably, CCL2 (MCP-1) recruits monocytes and macrophages to the wound site, facilitating their differentiation into M2 macrophages that promote tissue repair and mitigate inflammation. This transition from the inflammatory phase to the proliferative phase of wound healing is essential for efficient recovery [48]. CXCL1 (GRO) and CXCL10 (IP-10) are critical chemokines during the early stages of wound healing. CXCL1 is known for its role in recruiting neutrophils to the wound site, where they combat infection and initiate the inflammatory response. Conversely, CXCL10 facilitates the recruitment of T cells and macrophages, modulating the immune response to enhance tissue repair. Together, these chemokines create a conducive environment for wound healing by orchestrating the activities of key immune cells [49,50,51]. Interestingly, a previous study demonstrated that dental-pulp stem cells accelerated wound healing though CCL2-induced M2 macrophage polarization [52]. In addition, exfoliated deciduous teeth-derived MSCs secreted CSF1 (M-CSF) to support the survival, proliferation, and differentiation of macrophages, which modulated keratinocyte migration, enhanced viability, and promoted tissue repair [53,54]. Collectively, these results indicate that the secretome of hCV-MSCs contains a diverse array of ECM proteins and cytokines that enhance regenerative processes, including angiogenesis, cell migration, proliferation, and differentiation. Furthermore, their immunomodulatory effects foster a favorable environment for tissue regeneration. These dual capabilities position hCV-MSCs as promising candidates for therapeutic interventions in cutaneous wound healing. Of note, earlier time points, particularly during the inflammatory phase, are ideal for evaluating the immune-modulatory effects of the secretome, such as cytokine dynamics and immune-cell infiltration. Future studies should aim to include these time points to provide a more comprehensive understanding of the healing dynamics.

Our study presents a novel therapeutic strategy by combining local administration of hCV-MSC-laden hydrogel with systemic delivery of their secretome, demonstrating the synergistic enhancement of cutaneous wound healing (Figure 4, Figure 5, Figure 6 and Figure 7). This dual approach leverages the distinct yet complementary mechanisms of cellular and cell-free therapies. The local implantation of hCV-MSCs within a PEGDA/SA/Col-I hydrogel facilitates sustained paracrine signaling at the wound site, while the systemic administration of the secretome provides a concentrated bolus of bioactive factors that can amplify regenerative processes across multiple healing phases. This strategy effectively bridges the limitations of single-modality therapies. By supplementing locally engrafted cells with systemic secretome, we ensure the continuous delivery of critical factors even if MSC viability declines over time. The rationale for systemic secretome administration is supported by our proteomic data, which identified key angiogenic factors and immunomodulatory factors that may work synergistically with locally delivered hCV-MSCs to enhance vascularization and reduce inflammation. Moreover, our findings align with emerging trends in regenerative medicine, where combination therapies utilizing MSCs and their derivatives (e.g., exosomes, conditioned media) have demonstrated superior efficacy in preclinical models of myocardial infarction and spinal cord injury compared to standalone treatments [55,56]. To our knowledge, this study is the first to illustrate the additive benefits of combining placental MSC transplantation with systemic secretome administration in cutaneous wound healing, paving the way for clinically translatable regimens that optimize both local and systemic regenerative responses.

## 4. Materials and Methods

### 4.1. Cell Culture

Human chorionic villus mesenchymal stem cells (hCVMSCs) were isolated from full-term placentas obtained from three health donors, following approval by the Institutional Research Ethics Committee (ethical approval code: CREC 2020.313). Chorionic villi tissues were dissected from placentas, rinsed thoroughly with phosphate-buffered saline (PBS) to remove residual blood, and minced into fine fragments (approximately 1–5 mm^3^) using sterile scissors. The minced tissues were enzymatically digested with Dispase (1 U/mL; Stemcell Technologies, Vancouver, BC, Canada) and collagenase B (0.1%; Roche, Basel, Switzerland) at 37 °C for 1 h with gentle agitation. Subsequently, digested chorionic villi fragments were cultured in Dulbecco’s Modified Eagle Medium (DMEM; Gibco, Waltham, MA, USA) supplemented with 10% fetal bovine serum (FBS) and 1% penicillin/streptomycin at 37 °C in a humidified atmosphere containing 5% CO_2_. After 7–10 days of culture, adherent hCVMSCs were harvested and expanded upon reaching 80–90% confluence. Cells between passages 3 and 10 were used in subsequent experiments.

### 4.2. Flow Cytometry

The characterization of hCVMSCs was performed using the Human MSC Analysis Kit (BD Biosciences, San Jose, CA, USA) according to the manufacturer’s instructions. Briefly, cells were detached, washed, and centrifuged at 200× *g* for 3 min. Cell pellets were resuspended in staining buffer containing antibodies against CD105, CD90, CD73, CD44, CD45, CD34, CD11b, CD19, and HLA-DR at a 1:20 dilution and incubated on ice for 30 min. After two washes, labeled cells were resuspended in staining buffer and analyzed using a BD LSR Fortessa Cell Analyzer (BD Biosciences, San Jose, CA, USA). Data analysis was performed using FlowJo software (v10.2).

### 4.3. Multilineage Differentiation

The multilineage differentiation potential of hCVMSCs was evaluated at passage 3. Cells were seeded in 6-well plates and cultured until reaching approximately 70–80% confluence. Differentiation toward adipogenic, osteogenic, and chondrogenic lineages was induced using commercially available differentiation kits (Gibco, Waltham, MA, USA). After 2–3 weeks of induction, differentiated cells were assessed by specific staining methods: adipogenic differentiation was confirmed by staining lipid droplets with 0.2% Oil Red O solution (Sigma-Aldrich, St. Louis, MO, USA) for 5 min; osteogenic differentiation was detected by staining calcium deposits with 2% Alizarin Red solution (Sigma-Aldrich) for 20 min; and chondrogenic differentiation was visualized by staining proteoglycans with 1% Toluidine Blue solution (Sigma-Aldrich) for 30 min. Stained cells were imaged using a Nikon Ti-2 inverted fluorescence microscope (Nikon Corporation, Tokyo, Japan) to document differentiation outcomes.

### 4.4. Preparation of Stem Cell Secretome

Conditional media (CM) of hCVMSCs were prepared following our previously established protocol with slight modifications [32]. Briefly, hCVMSCs were seeded in 75 cm^2^ flasks and cultured in complete growth medium until 80–90% confluence. Cells were then washed three times with PBS and incubated in 20 mL serum-free DMEM for 48 h. Subsequently, CM was collected, centrifuged at 2000× *g* for 10 min to remove cellular debris, and concentrated. The concentrated CM was dialyzed against double-distilled water at 4 °C to remove ions and molecules smaller than 1.0 kDa and then lyophilized (freeze-dried). Protein concentrations in the secretome samples were quantified at a wavelength of 562 nm using a Pierce™ BCA Protein Assay Kit (Thermo Fisher Scientific, Waltham, MA, USA).

### 4.5. Cell Viability Assay

Cell viability and proliferation were assessed using the MTT assay (Sigma-Aldrich). Briefly, hCVMSCs at passage 3 were seeded into 96-well plates at a density of 1.5 × 10^3^ cells per well and cultured under standard conditions. At predetermined time points, 10 µL MTT solution was added to each well containing 100 µL culture medium, and cells were incubated at 37 °C and 5% CO_2_ for 4 h. After incubation, the medium was replaced with 100 µL dimethyl sulfoxide (DMSO; Santa Cruz Biotechnology, Dallas, TX, USA) to dissolve the formazan crystals. Plates were protected from light and gently shaken for 15 min at 100 rpm. Absorbance was measured at 570 nm using a microplate reader.

### 4.6. Mass Spectrometry Sample Preparation and Analysis

Secreted proteins from CM samples were extracted and prepared for mass spectrometry analysis using the EasyPep Mini MS Sample Prep Kit (Thermo Fisher Scientific) according to the manufacturer’s instructions. Briefly, protein samples (50 µg) were reduced, alkylated, and enzymatically digested with sequencing-grade trypsin for 3 h at 37 °C. Digested peptides were desalted using C18 peptide clean-up columns and dried by vacuum centrifugation prior to analysis. Fractionation and liquid chromatography–tandem mass spectrometry (LC-MS/MS) analyses were performed at the University Research Facility in Chemical and Environmental Analysis, The Hong Kong Polytechnic University. Peptides were separated using an Acclaim PepMap RSLC analytical column coupled to a Dionex Ultimate 3000 RSLCnano system and analyzed on an Orbitrap Fusion Lumos mass spectrometer (Thermo Fisher Scientific) operated in data-dependent acquisition mode. Survey scans were obtained over an *m*/*z* range of 350–1500 at a resolution of 500,000 FWHM, followed by MS/MS of the most intense precursor ions with charge states between 2 and 4. Proteomic data analysis was conducted using Progenesis QI software (V2.3, Nonlinear Dynamics, Newcastle Upon Tyne, UK), and identified peptides were searched against the Swiss-Prot human database, allowing for methionine oxidation as a variable modification. Proteins identified with ≥1 unique peptides across all samples were considered abundant.

### 4.7. Preparation of PEGDA/SA/Col-I Hydrogel

Sodium alginate (SA; medium viscosity, Sigma-Aldrich, USA) powder was sterilized by ultraviolet (UV) irradiation and dissolved in PBS at 2% (*w*/*v*). This SA solution was then further sterilized using a 0.22 μm filter (Millipore, Billerica, MA, USA) before use. Poly (ethylene glycol) diacrylate (PEGDA; molecular weight: 1 kDa, EFL, Suzhou, China) was dissolved at 12% (*w*/*v*) in lithium phenyl-2,4,6-trimethylbenzoyl phosphinate (LAP; 0.2% *w*/*v* in PBS) solution. Both SA and PEGDA solutions were further sterilized by filtration through a 0.22 μm filter (Millipore) prior to use. Human type I collagen solution (Col-I; Corning, Corning, NY, USA) was diluted in PBS at a ratio of 1:20 before use. To prepare the composite hydrogel, SA solution (100 μL), PEGDA solution (100 μL), and diluted Col-I solution (20 μL) were homogeneously mixed via a sterile T-branch connector and subsequently crosslinked by exposure to UV light (λ = 365 nm) for 60 s. After UV crosslinking, secondary ionic crosslinking was performed by adding 100 mM CaCl_2_ solution for 60 s. The resulting PEGDA/SA/Col-I hydrogel was rinsed thoroughly with PBS before subsequent use.

### 4.8. Fabrication of hCVMSC-Laden PEGDA/SA/Col-I Hydrogel

To fabricate cell-encapsulated hydrogels, PEGDA, SA, diluted collagen I solutions, and hCVMSC suspension (initial cell density: 1 × 10^9^ cells/mL) were uniformly mixed at a volume ratio of 100:100:20:2, resulting in a final cell density of approximately 1 × 10^7^ cells/mL. Then, 100 μL aliquots of the cell-containing mixture were pipetted into individual wells of a 6-well plate and crosslinked first by UV irradiation (365 nm, 10 mW/cm^2^) for 1 min, followed by secondary ionic crosslinking using 1 mL of 100 mM CaCl_2_ solution for an additional minute. After crosslinking, hydrogels were rinsed three times with PBS to yield cell-laden PEGDA/SA/Col-I hydrogels. To evaluate cell proliferation, smaller aliquots (5 μL) of cell-laden hydrogel mixture were similarly crosslinked in 96-well plates. Following washing, each hydrogel was cultured in 100 μL complete growth medium for up to 10 days. Cell viability and proliferation were quantitatively assessed using the MTS assay at days 0, 4, 7 and 10.

### 4.9. Live/Dead Assay

The viability of hCVMSCs encapsulated within PEGDA/SA/Col-I hydrogels was qualitatively assessed at days 0 and 3 using a Live/Dead Viability/Cytotoxicity Kit (Invitrogen, Carlsbad, CA, USA). Following three PBS washes, hydrogels were incubated with 2 μM calcein-AM (live-cell indicator) and 4 μM Ethidium homodimer-1 (EthD-1; dead-cell indicator) for 30 min at room temperature in the dark. Fluorescence images were captured using a Nikon Ti-2 inverted fluorescence microscope to visualize and document cell viability.

### 4.10. Protein Secretion Assay

A total of 100 μL of hCVMSCs-laden PEGDA/SA/Col-I hydrogel was plated in each well of 6-well plates and incubated for 24 h. After incubation, the culture medium was removed, and the cell-laden hydrogels were washed three times with serum-free DMEM. Then, 2 mL of serum-free DMEM was added to each well. After 48 h, the medium was collected and centrifuged at 2000× *g* for 10 min to pellet debris. The concentration of secreted proteins was quantified using a Pierce BCA Protein Assay Kit (Thermo Fisher Scientific, Waltham, MA, USA), with measurements taken at 562 nm.

### 4.11. Cutaneous Wound Healing Model in Rats and Treatment Strategy

All animal procedures were approved by the Laboratory Animal Experimentation Ethical Committee of the Chinese University of Hong Kong and conducted in accordance with the Guidelines for the Care and Use of Laboratory Animals. Male Sprague Dawley (SD) rats (8–12 weeks old) were acclimated under standard laboratory conditions (12/12 h light/dark cycle, 22 °C, 50–60% humidity) for at least one week prior to surgery. Full-thickness excisional wounds were created under isoflurane anesthesia using 10 mm circular biopsy punches (Integra Miltex) applied to the depilated and disinfected dorsal skin. Two experimental batches were conducted. In the first batch, rats were assigned to three groups: the PBS group (100 μL sterile PBS applied topically), the hydrogel group (100 μL hydrogel applied directly on the wound), and the hCVMSC + Gel group (100 μL hydrogel containing 1 × 10^6^ hCVMSCs applied directly on the wound). Wound areas were measured on days 0, 3, 5, 7, 10, and 20 using a 10 mm silicone template. In the second batch, rats were divided into four groups: PBS, hydrogel, hCVMSC + Gel, and hCVMSC + Gel + secretome, where the latter received 100 μL hydrogel with hCVMSCs applied locally combined with secretory factors injected via tail vein. Treatments were administered immediately after wound creation and repeated one week later, with wound measurements taken on days 0, 3, 7, 10, 15, and 20. Images were analyzed using ImageJ software (1.54), and wound closure was calculated as a percentage of the original wound area. The area under the curve (AUC) for each wound was normalized to the mean AUC of the vehicle control group.

### 4.12. Histology, Immunohistochemistry, and Immunofluorescence

On day 20, animals were euthanized via carbon dioxide exposure followed by cervical dislocation. Full-thickness dorsal skin was collected, fixed in 4% paraformaldehyde, and embedded in paraffin. Tissue sections (5 µm) were prepared for histological and immunofluorescence analyses, including hematoxylin and eosin (H&E), Masson’s trichrome, and immunofluorescence staining.

For IF staining, antigen retrieval was conducted using citrate buffer (10 mM sodium citrate, 0.05% Tween-20, pH 6.0) by microwaving at high power for 3 min, followed by steaming at 95 °C for 15 min. Sections were blocked for 1 h at room temperature, followed by overnight incubation at 4 °C with primary antibodies: rabbit monoclonal anti-KRT14 (1:200; Proteintech, Rosemont, IL, USA), mouse monoclonal anti-KRT10 (1:200; Invitrogen, USA), rat monoclonal anti-CD31 (1:200; Abcam, Cambridge, UK), and rabbit polyclonal anti-IL6 (1:100, ImmunoWay, San Jose, CA, USA). After washing, sections were incubated for 1 h at room temperature with appropriate secondary antibodies: Alexa Fluor 568 donkey anti-rabbit IgG, Alexa Fluor 488 donkey anti-mouse IgG, or Alexa Fluor 594 donkey anti-rat IgG (all at 1:400 dilution; Invitrogen, USA). Nuclei were counterstained with DAPI. Fluorescence images were captured using a Leica TCS SP8 inverted confocal microscope, employing consistent acquisition parameters across all samples for quantitative comparisons.

Quantitative analyses were performed using ImageJ (Fiji) software (1.54). Regions of interest (ROIs) were defined based on anatomical structures visualized by DAPI staining. Positive cells were identified using a uniform intensity threshold applied consistently across all images. Granulation tissue gaps were measured using ImageJ’s freehand selection tool to outline and quantify the gap area. For collagen deposition analysis, Masson’s trichrome-stained images were color-separated using ImageJ to isolate collagen fibers (dark blue). The collagen-positive stained area was quantified and expressed relative to the total tissue area for comparison among groups.

### 4.13. Statistical Analysis

Statistical analyses were performed using GraphPad Prism software (version 9.0; GraphPad Software, La Jolla, CA, USA). Comparisons between two groups were conducted using a two-tailed Student’s *t*-test. Comparisons among multiple groups were assessed using one-way analysis of variance (ANOVA), followed by Tukey’s multiple-comparison post hoc test. Post hoc test results are reported only if overall significance was detected by ANOVA. Data are expressed as mean ± standard deviation (SD). Statistical significance was defined as *p* < 0.05.

## Data Availability

The proteomics data has been deposited in MassIVE doi:10.25345/C5F47H56X.

## Supplementary Materials

The following supporting information can be downloaded at: https://www.mdpi.com/article/10.3390/ijms26146888/s1.

## Abbreviations

ANOVA	one-way analysis of variance
ANXA1	annexin A1
AUC	Area Under the Curve
CCL2	C-C Motif Chemokine Ligand 2
CM	conditional media
CSF1	Colony-Stimulating Factor 1
ECM	Extracellular Matrix
FBS	Fetal Bovine Serum
FN1	Fibronectin 1
GO	Gene ontology
GRO	Growth-Regulated Oncogene
hCV-MSCs	Human Chorionic Villus Mesenchymal Stem Cells
IL	Interleukin
ITGB1	integrin beta-1
KRT14	keratin 14
KRT10	keratin 10
MMPs	Matrix Metalloproteinases
MSC	Mesenchymal Stem Cell
PAI-1	Plasminogen Activator Inhibitor-1
PBS	phosphate-buffered saline
PEGDA	Poly (ethylene glycol) Diacrylate
ROI	region of interest
SA	Sodium Alginate
SERPINE1	serpin family E member 1
TNF-α	Tumor Necrosis Factor-alpha
WNT5A	Wingless Int-1 Family Member 5A
