# Supplementary material for: Combination Therapy with Human Chorionic Villi MSCs and Secretory Factors Enhances Cutaneous Wound Healing in a Rat Model
Source: Int J Mol Sci. 2025 Jul 17;26(14):6888. doi: 10.3390/ijms26146888 (PMC12295468; doi:10.3390/ijms26146888)
Supplement: Supplementary file 1 [file ijms-26-06888-s001.zip › Supplementary Figure Captions.pdf]

Supplementary Figures

Figure S1. Morphology of hCV-MSCs. (A) Phase-contrast images of hCVMSCs derived from 3 donors. (**B)** Cell proliferation of hCV-MSCs was assessed by MTT assay. Experiments were repeated at least three times. **,$$,## $p < 0.01$, ***,$$$,### $p < 0.001$, ****,$$$$,#### $p < 0.0001$

Figure S2. Characterization of hCV-MSCs. (A) Flow cytometry analysis of hCV-MSCs using positive markers, CD90, CD105, CD44 and CD73 and negative markers, CD34, CD11, CD19, CD45 and HLA-DR. (**B)** hCV-MSCs could undergo osteogenic, adipogenic and chondrogenic differentiation as demonstrated by Alizarin red, Oil red O, and Alcian blue staining. Experiments were repeated at least three times.

Figure S3. PEGDA/SA/Col-I hydrogels are biocompatible with hCV-MSCs. (A) Live/Dead fluorescent images of hCV-MSCs cultured within PEGDA/SA/Col-I hydrogel. **(B)** MTS assay results determining the cell viability of hCV-MSCs encapsulated in the PEGDA/SA/Col-I hydrogel for 10 days. (**C)** Protein secretion assay showing the concentration of protein secreted by hCV-MSCs encapsulated in PEGDA/SA/Col-I hydrogel. ** $p < 0.01$.
