# Supplementary figures and images for: Combination Therapy with Human Chorionic Villi MSCs and Secretory Factors Enhances Cutaneous Wound Healing in a Rat Model
Source: Int J Mol Sci. 2025 Jul 17;26(14):6888. doi: 10.3390/ijms26146888 (PMC12295468; doi:10.3390/ijms26146888)

**A**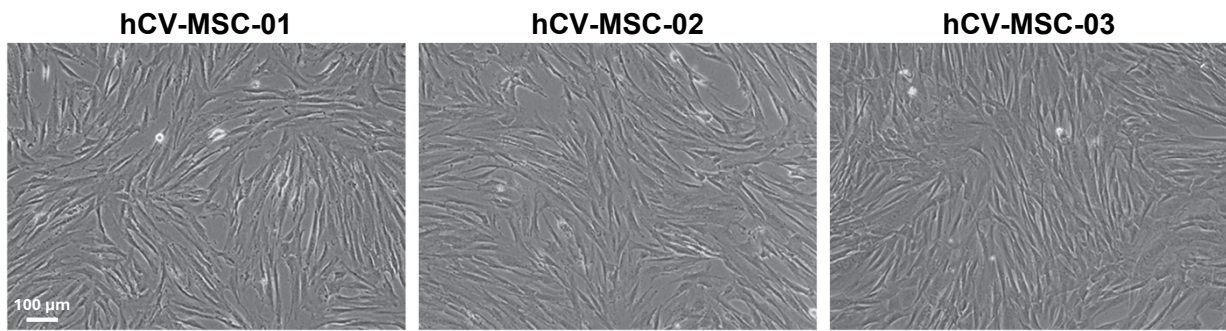**B**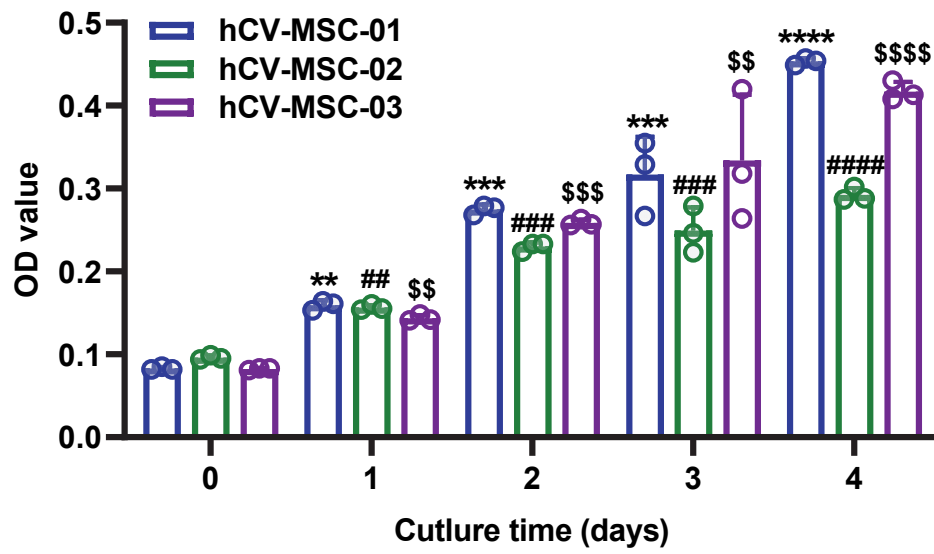

**A**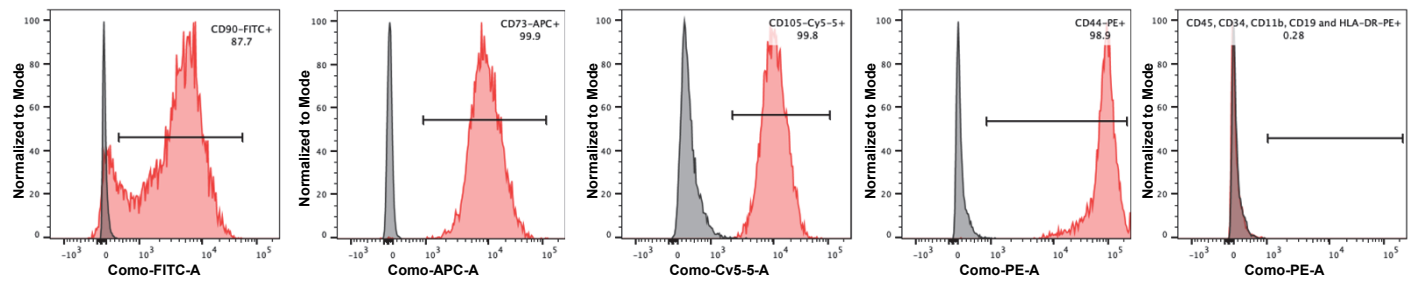**B**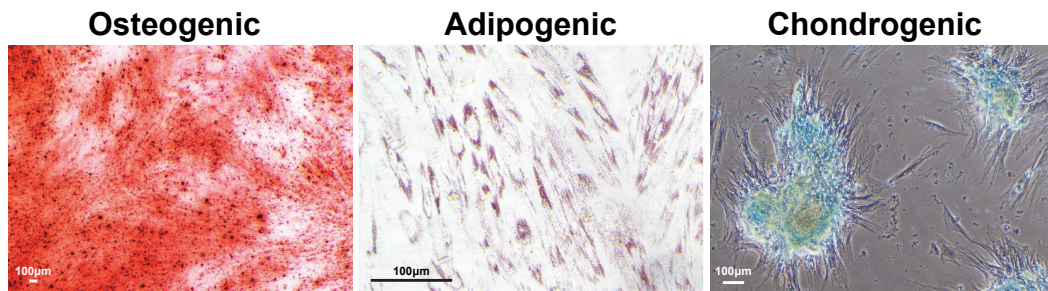

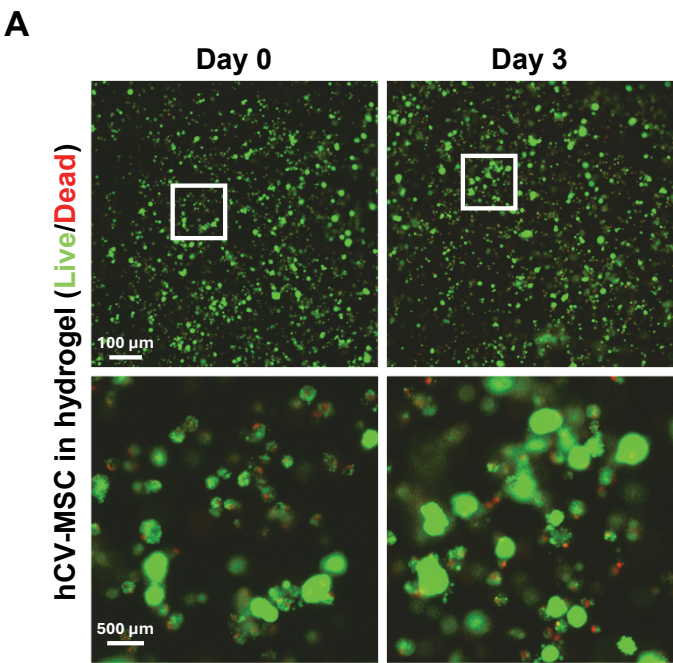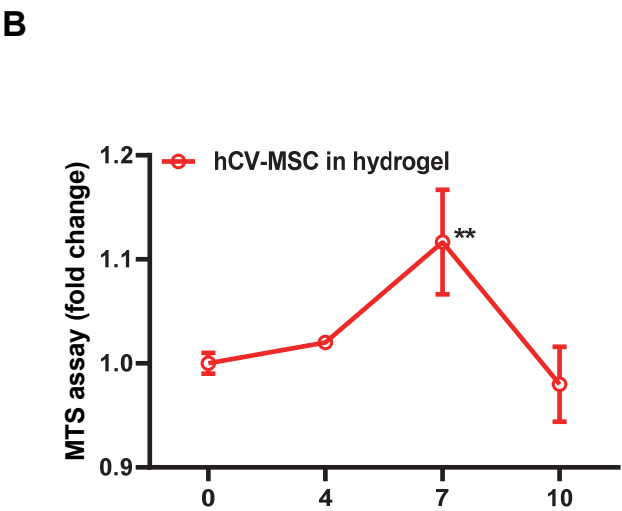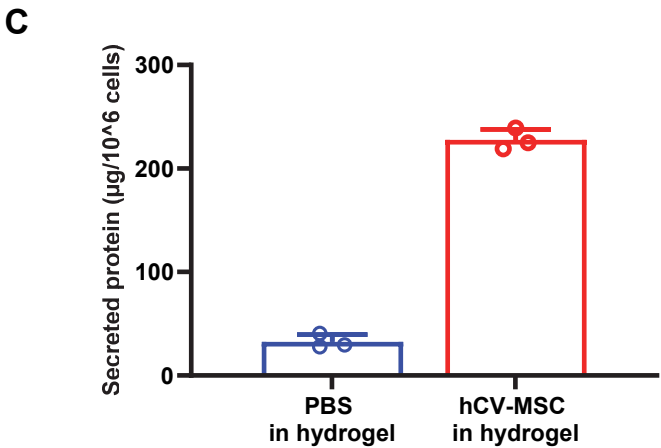

Supplement: Supplementary file 1 [file ijms-26-06888-s001.zip › Supplementary Figures.pdf]
